# The effects of modified problem-solving therapy on depression, coping, and self-efficacy in elderly nursing home residents

**DOI:** 10.3389/fpsyg.2022.1030104

**Published:** 2023-01-06

**Authors:** Xiaoqi Wu, Jie Li, Chun Zhang, Xing Zhou, Xiaoqian Dong, Huan Cao, Yinglong Duan, Sha Wang, Min Liu, Qiuxiang Zhang, Jianfei Xie

**Affiliations:** ^1^Nursing Department, The Third Xiangya Hospital of Central South University, Changsha, China; ^2^Xiangya Nursing School, Central South University, Changsha, China

**Keywords:** modified problem-solving therapy, nursing home, the elderly, depression, coping ability, self-efficacy

## Abstract

**Background:**

With the increasing trend of aging, the mental health problems of the elderly require urgent attention. Depression is a common psychological problem of the elderly, which affects their quality of life and physical health. Problem-solving therapy can effectively improve depression in the elderly, but there are few studies on problem-solving therapy for depression in the elderly in China. The purpose of this study was to evaluate the effects of modified problem-solving therapy (MPST) on depression, coping and self-efficacy in elderly nursing home residents.

**Methods:**

This study was a randomized controlled trial. A total of 60 older adults from two nursing homes were recruited to participate in this study and randomly assigned to the intervention group (MPST) or the control group (usual care). The intervention lasted 8 weeks, and information on depression, coping skills, and self-efficacy was collected before the intervention, immediately after the intervention, and 3 months after the intervention. Repeated measures ANOVA was used to compare changes at multiple time points between the two groups. If the interaction effect (group * time) was significant, independent samples t-test was used to compare the differences in outcome indicators between groups at post-intervention and 3 months post-intervention.

**Results:**

Compared to the control group, depression scores in the intervention group were significantly lower at the end of the intervention and remained significantly lower than the control group 3 months post-intervention (*p* < 0.05). Negative coping and self-efficacy in the intervention group also improved significantly at the end of the intervention, and 3 months post-intervention, while positive coping in the two groups did not differ significantly at 3 months post-intervention.

**Conclusion:**

The findings of this study suggest that MPST could be beneficial in reducing depressive symptoms and enhancing positive coping and self-efficacy levels in older adults in nursing homes.

## 1. Introduction

The number of people over 60 years old in China reached 267 million in 2021, of which 201 million (14.2%) are over 65, which means that China’s aging problem has already become severe (National Bureau of Statistics of China, 2022). As a part of China’s long-term care system, nursing homes assume an enormous role in developing healthy aging. Due to the late development of China’s nursing home system, most nursing homes are facing several problems (lack of professional caregivers, security service system to be improved, etc.), and their services are mostly daily care and medical services, lacking psychological care services (Chen et al., 2021; Meng et al., 2021). Additionally, the closed environment of nursing homes and the lack of social support for residents also influence negatively on the mental health of elderly in nursing homes (Zhao et al., 2018). With aging, older adults experience degenerative changes of the nervous system, including sensory slowing, attention decline, and thinking slowdown, which may result in low self-esteem, low self-efficacy and negative coping, heightening the risk of depression in the elderly (Fernández-Pérez et al., 2020; Whitehall et al., 2020). A meta-analysis showed that the overall prevalence of depressive symptoms among older adults in nursing homes in China was up to 37.49% (Zhao et al., 2020). Depression in older adults is not only associated with physical health, quality of life, and suicide risk, but also a potentially increased social burden (Casey, 2017; Alexopoulos, 2019). Hence, implementing psychological interventions to reduce depressive symptoms among the elderly in nursing homes is of great value.

As a non-pharmacological treatment method, problem-solving therapy (PST) is structured, systematic, and widely applicable, and has been widely used in the treatment of depressed patients with effective results (Cuijpers et al., 2018). PST is a problem-oriented approach to guide depressed patients to identify their problems, supplemented by problem-solving skills correctly (e.g., setting reasonable goals, brainstorming methods to identify solutions). Therapists use PST to help individuals better manage their life problems and improve their daily life experiences, thus effectively solving problems, improving their coping skills and reducing depressive symptoms caused by complex problem-solving (Alexopoulos et al., 2016; Shang et al., 2021). Alexopoulos et al. (2016) conducted a randomized controlled trial of problem-solving therapy versus supportive psychological interventions in homebound elderly depressed patients and showed that problem-solving therapy was superior to supportive psychological interventions in improving depression in the elderly.

Unlike other psychotherapies, PST can be administered by healthcare professionals with non-psychological backgrounds, such as community physicians, nurses, and social workers who can qualify as therapists after a brief training period. Two studies that took nurses as therapists to intervene with depressed elderly patients showed that nurse-led PST had an ameliorating effect on depression (Gellis et al., 2014; Haejung et al., 2015). This characteristic of PST enabled a much lower intervention cost and facilitated its replication. Compared to other psychological interventions, PST has greater adaptability to the environment, and is not only suitable for face-to-face interventions but also performed in telephone format, for which different forms of PST could improve the patients’ depression (Kirkham et al., 2016). Despite its many advantages, PST is rarely used in the elderly population in China, but it has been carried out in other populations (kidney transplant patients and stroke patients) with positive results, which have confirmed the improvement of depressive symptoms of PST, indicating the applicability of problem-solving therapy in the depressed population in China (Gong, 2015; Ye et al., 2019).

**Figure 1:**
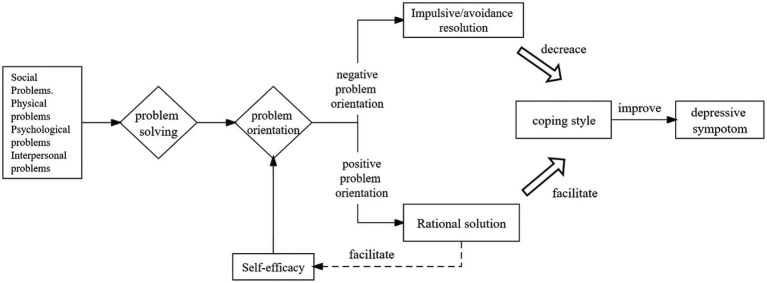
Theoretical framework.

Based on previous observations by the team members, the researchers found that the elderly in nursing homes have low knowledge towards depression and psychological interventions, and the traditional PST program has a relatively short time for education on depression. Thus, a modified problem-solving therapy (MPST) in this study was designed based on social problem-solving theory and self-efficacy theory (Bandura, 1977; D’Zurilla and Nezu, 1990; Chang and D’Zurilla, 1996) see Figure 1. The traditional PST, and the main differences between MPST and traditional PST are (1) increasing the time for older adults to understand the knowledge about depression and intervention. In the first session, only the knowledge was introduced. Only introducing the relevant knowledge in the first session is beneficial for the older adults to adequately understand the relevant knowledge and increase the interaction between the researcher and older adults, deepening older adults’ trust in the researcher. (2) By adding a group intervention, we encourage older adults to share their experiences and feelings during the intervention to increase their recognition and sense of belonging, and improve their confidence in coping with problems. The study aimed to explore the effects of modified problem-solving therapy (MPST) on depression, coping and self-efficacy in nursing homes elderly. The main hypothesis of this study was that participants who received MPST would experience greater remission of depressive symptoms compared to those who received usual care. The secondary hypothesis was that participants who received MPST had higher positive coping and self-efficacy.

## 2. Materials and methods

### 2.1. Study design

The study design was a randomized controlled trial from two nursing homes in Changsha City, China, between March and December 2021. The researchers contacted the directors of the nursing home and obtained their consent to gain information about the living conditions of the elderly. The researchers introduced the research purpose to eligible elderly depressed people in nursing homes to obtain informed consent and recruited participants based on inclusion and exclusion criteria. After participants provided informed consent and completed the baseline questionnaire (T1), participants were assigned to the intervention group (MPST) and control groups (usual care) according to a random number table. The following measurements were made at the immediate post-intervention (T2) and 3 months post-intervention (T3) to analyze the effect of the intervention.

### 2.2. Participants

The inclusion criteria of the participants were (1) Chinese-speaking adults (aged ≥60 years); (2) screened positive for depression (score > 5 using the Brief Geriatric Depression Scale); (3) able to read and communicate in daily life; (4) volunteered to participate in this study. Participants were excluded if they had (1) severe cognitive impairment or serious physical illnesses such as cardiopulmonary failure; (2) currently participating in other studies; (3) currently undergoing psychotherapy or taking antidepressant medication.

### 2.3. Sample calculation

The sample size was calculated by reference to Zvi’s study (Gellis et al., 2007), through the formula for the sample size required to compare the mean of two samples (Sun Zhengqiu, 2014).


n1=n2=2μα+μβδσ2+14μα2

A 5% level of significance (two-tailed test) and a power of 0.90 were adopted, *μ_α_* = 1.96 *μ_β_* = 1.28, *μ_α_* + *μ_β_* = 3.24, effect size =δσ = (μ_1_-μ_2_)/σ = (6.77–1.77)/5.14 = 0.973, *n*_1_ = *n*_2_ = 2(3.24/0.973)^2^ + $\frac{1}{4}1.96^{2}$≈23, considering a 20% sample attrition rate, 29 older adults in each group is sufficient to satisfy the hypothesis of the parametric test.

### 2.4. Randomization

The random grouping method of this study was as follows: Firstly, random numbers were constructed by SPSS 19, and the fixed value of 100 was set as the starting point. The range of random numbers was within the range of 1 to 60, and random numbers were divided into group 1 and group 2 according to the visual box. Group 1 was set as the intervention group and group 2 as the control group. Based on the selected list of the elderly in nursing homes, they were sorted from 1 to 60 in the initial order of last name and matched with the random numbers constructed by SPSS 19.0. If the number was 1, it was included in the intervention group, and if it was 2, it was be included in the control group, so as to achieve randomization.

### 2.5. Intervention

#### 2.5.1. Control group

The control group received routine care. That is, they were given the necessary care services by nursing home staff, including daily care services, medical care services, health education and social activities.

#### 2.5.2. Intervention group

The intervention group received MPST on the basis of routine care. The MPST was conducted for eight weekly sessions of 30 to 60 min. The MPST intervention program was designed by researchers working in the field of geriatric psychology, including three graduate students, one clinical psychologist, and two geriatric nursing specialists with more than 10 years of experience. Of the three graduate students, two were assigned to collect pre-and post-intervention data, the other graduate student was received training from the clinical psychologist on the content of the MPST and assigned to implement the intervention. The clinical psychologist administered the development of the intervention protocol and the training of the intervention implementers. Two geriatric nursing specialists were involved in the development of the intervention protocol. Based on the preliminary survey, researchers found that the elderly in nursing homes lacked knowledge about depression. Hence, this study developed an intervention model based on the traditional PST to be more suitable for the elderly in Chinese nursing homes. For example, the traditional PST had more content in the first session, including knowledge of depression, the introduction of PST and formal problem solving, while the first session of MPST only introduced the depression-related knowledge and the PST intervention steps to assure that the elderly adequately understood the depression-related knowledge and the content of the study protocol, and the second session formally performed problem solving; based on observations, the researchers found that communication needs existed among the elderly in nursing homes, while traditional PST rarely involved communication within the depressed participants. MPST in this study set up a group session with the purpose of sharing information about their difficulties and experiences during the intervention. Table 1 illustrates the themes of the eight-week MPST intervention. The entire intervention was led by one graduate student. Except for the seventh use of group intervention, MPST was delivered through a one-on-one, in-person approach. The first session focused on introducing the elderly to depression, guiding them to recognize the relationship between depression and daily life problems, and introducing the PST steps. In the second to sixth sessions, the elderly were provided with daily life cases to promote their awareness and selection of problems, and brainstorming techniques were used to create a problem-solving plan and activity schedule. In subsequent meetings, the researcher would be engaged in discussion with the elderly about the implementation of the program and the difficulties encountered in the course of action.

**Table 1:** Outline of MPST intervention.

Time	Session content	Assignment
Week 1	1. Introduction to depression	Be familiar with depression and PST
	2. Introduction to PST	
Week 2	1. Review problem solving therapy	1. Deepen the knowledge of PST-related steps
	2. Formally apply the PST steps: select a problem, set a goal, generate multiple solutions, select the best solution, and develop an activity schedule based on the solution	2. Complete activity schedule form
	3. Incorporate personal interests of the elderly and add activities to the schedule that will promote their sense of enjoyment	
Week 3–6	1. Evaluate the completion of last week’s activity schedule	Complete activity schedule form
	2. Evaluate solution implementation and effectiveness	
	3. Select new issues for PST	
Week 7	1. Organize a group of 4–6 people	Complete activity schedule form
	2. Older adults share their feelings and experiences of the intervention process	
	3. Complete a new schedule of activities	
Week 8	1. Review the steps of PST	Summarizing intervention experience
	2. Summarize the entire intervention process	

### 2.6. Measures

#### 2.6.1. Demographics

Demographic and disease information such as gender, age, education level, marital status, time in the nursing home, and type of disease were collected.

#### 2.6.2. Depression

The short version of the Geriatric Depression Scale (GDS-15) was used to evaluate depression, which had 15 items (Sheikh and Yesavage, 1986). The total score ranges from 0 to 15, with a score of <6 being the normal range, 6–10 being mild depression, and 11–15 being moderate to severe depression. Previous study has reported that the Chinese version of the scale has a Cronbach’s alpha value of 0.79, with a retest reliability of 0.73, which indicates that the Chinese version GDS-15 is widely applicable to the Chinese elderly (Tang, 2013).

#### 2.6.3. Coping ability

The Brief Coping Style Scale was designed by Xie (1998) to evaluate coping ability, which had 20 items. The scale includes two dimensions: positive coping (items 1–12) and negative coping (items 13–20) and was rated on a four-point Likert scale from “not adopted” to “often adopted,” with scores ranging from 0 to 3. The positive and negative coping scores are the sums of the respective entries, and the scores range from 0 to 36 and 0 to 24, respectively. The reliability of the scale applied to the elderly population was good, with a Cronbach’s α value of 0.908, which had good reliability and internal consistency in Chinese populations (Zhu et al., 2016).

#### 2.6.4. Self-efficacy

The General Self-Efficacy Scale was used to evaluate self-efficacy with 10 items (Schwarzer et al., 1999). The scale is based on a four-point Likert scale, ranging from “not at all correct” to “completely correct,” with scores ranging from 1 to 4, and the scale’s total score is the sum of the entries. The higher the total score, the higher the patient’s self-efficacy level. This scale is a commonly used self-efficacy scale for the elderly at home and abroad, and the Chinese version of the scale has a Cronbach’s alpha value of 0.81, which is suitable for screening self-efficacy of the Chinses elderly (Qin et al., 2020).

All scale data before and after the intervention were collected by two graduate students, who received training on the questionnaire-related items, including the requirements for questionnaire administration and completion; the purpose and significance of the study.

#### 2.6.5. Ethics approval

The study was formally approved by the Review Board of Xiangya School of Nursing, Central South University (No. E202179). This research was conducted after obtaining informed consent from participants. The researchers made an effort to ensure that the participants were fully informed about the aims of this study, the process, and the potential risks and benefits. For those whose depressive symptoms did not decrease after receiving the MPST, the researchers offered other psychological treatment options for them. If the elderly in the control group had intervention needs at the end of the study, they were provided with the same quality of intervention support as the intervention group.

#### 2.6.6. Statistical analysis

SPSS 19.0 software was used for data analysis. (1) Descriptive statistical analysis: Normality test was performed for the quantitative data in general information, depression score, coping style and self-efficacy score; mean and standard deviation were used for data conforming to normal distribution, median and interquartile spacing were used for data not conforming to normal distribution; frequency and percentage were used for qualitative data. (2) Statistical inference: independent sample t-test and chi-square test were used to compare baseline information between the intervention and control groups; Group effects, time effects, and interaction effects (group * time) were calculated by repeated measures analysis of variance (ANOVA) to examine the mean score differences on outcome measures within and between the groups at baseline, post-intervention and 3 months post-intervention. If the interaction effect (group * time) was significant, independent samples t-test was used to compare the differences in outcome indicators between groups at post-intervention and 3 months post-intervention. The test level *α* = 0.05 and *p* < 0.05 indicates statistical significance.

## 3. Results

**Figure 2:**
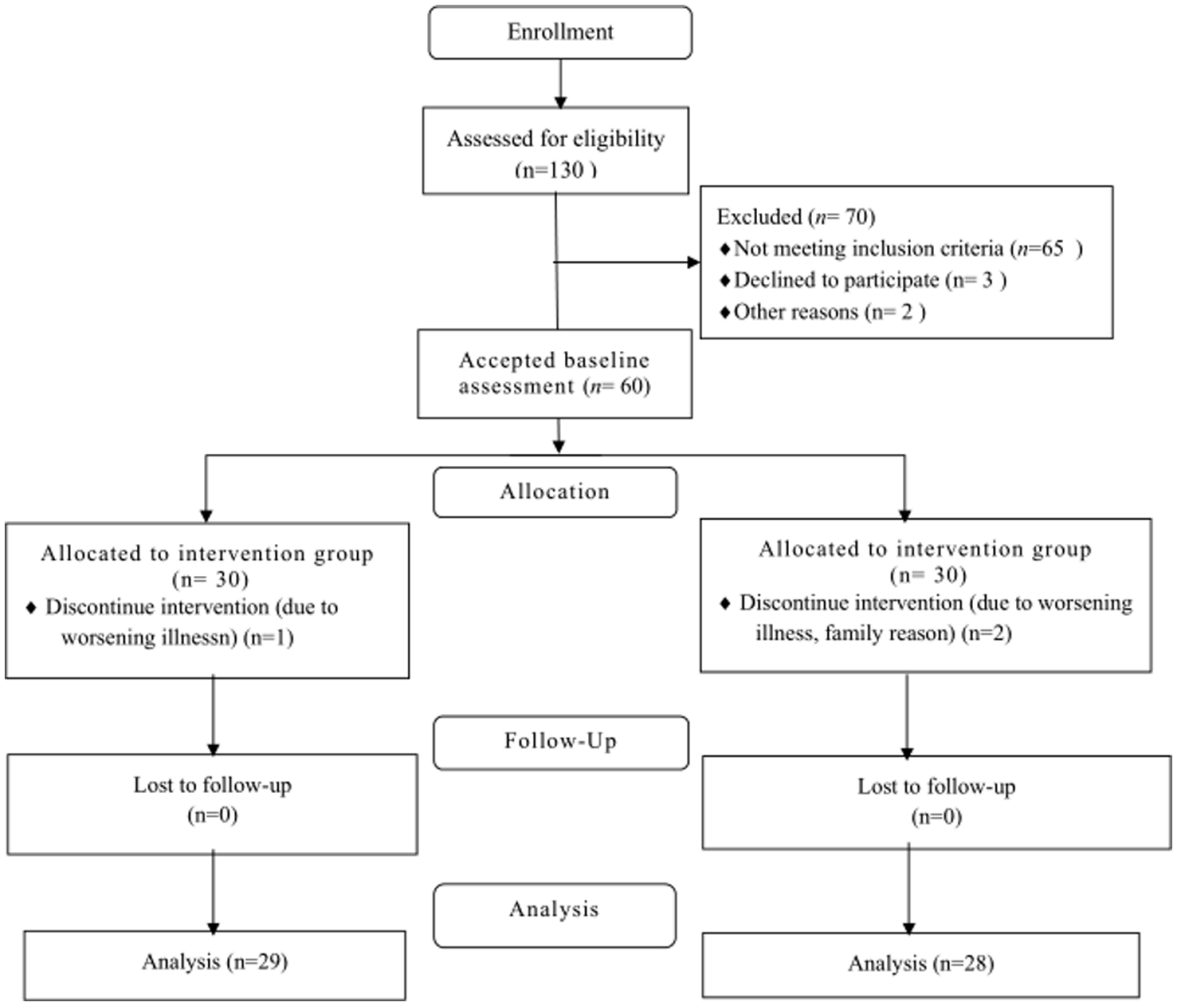
Flow chart of study participants.

Sixty older adults participated in this study, and three older adults were lost during the intervention period, including one person in the intervention group (*n* = 29) and two persons in the control group (*n* = 28), for a total of 57 older adults who completed the entire intervention. The reasons for the loss of visit: one older person in the intervention group was hospitalized and withdrew due to aggravation, one adult in the control group went home and one adult was hospitalized and withdrew, as shown in Figure 2.

### 3.1. Baseline measurements

No statistical differences were found between the intervention and control groups in age, sex, educational status and other sociodemographic data (see Table 2). There was no significant difference in the scores of depression, positive coping, negative coping and self-efficacy between the intervention group and the control group (see Table 3).

**Table 2:** Baseline characteristics of participants.

	Intervention group *n* = 30	Control group *n* = 30	*t/χ* ^2^	*P*
Age (M ± SD)	84.23 ± 7.07	83.40 ± 7.29	0.449	0.655
Gender (*n*, %)				
Male	9 (30.0%)	7 (23.3%)	0.341	0.559
Female	21 (70.0%)	23 (76.7%)		
Education (*n*, %)				
Junior high school or below	5 (16.7%)	10 (33.3%)	2.424	0.298
High school	6 (20.0%)	6 (20.0%)		
College degree or above	19 (63.3%)	14 (46.7%)		
Number of children (M ± SD)	2.37 ± 1.07	2.20 ± 1.03	0.616	0.541
Visit frequency by children (*n*, %)				
<1 time/month	5 (16.7%)	7 (23.3%)	3.167	0.367
1 time/month	8 (26.7%)	4 (13.3%)		
2–4 times/month	13 (43.3%)	11 (36.7%)		
>5 times/month	4 (13.3%)	8 (26.7%)		
Number of diseases (M ± SD)	2.97 ± 0.62	2.63 ± 0.77	1.860	0.068
Drug usage (*n*, %)				
Yes	28 (93.3%)	25 (83.3%)	0.647	0.421
No	2 (6.7%)	5 (16.7%)		
Participation in group activities (*n*, %)				
Yes	11 (36.7%)	15 (50.0%)	1.086	0.297
No	19 (63.3%)	15 (50.0%)		
Stay in a nursing home (*n*, %)				
<6 months	7 (23.3%)	6 (20.0%)	2.359	0.501
6–12 months	3 (10.1%)	7 (23.3%)		
1–2 years	4 (13.3%)	5 (16.7%)		
>2 years	16 (53.3%)	12 (40.0%)		
Depression score(M ± SD)	9.40 ± 2.01	9.33 ± 2.37	0.118	0.907
Positive coping(M ± SD)	20.38 ± 3.61	20.68 ± 3.16	−0.332	0.741
Negative coping(M ± SD)	14.07 ± 2.91	14.37 ± 2.92	−0.399	0.692
Self-efficacy score(M ± SD)	24.50 ± 6.11	24.13 ± 5.99	0.235	0.815

**Table 3:** Depression, coping, and self-efficacy at baseline for both groups.

	Intervention group *n* = 29	Control group *n* = 28	*t/χ* ^2^	*P*
Depression score (M ± SD)	9.38 ± 2.04	9.25 ± 2.43	0.22	0.828
Positive coping (M ± SD)	20.38 ± 3.61	20.68 ± 3.16	−0.332	0.741
Negative coping (M ± SD)	14.21 ± 2.90	14.50 ± 2.98	−0.38	0.708
Self-efficacy score (M ± SD)	24.48 ± 6.22	24.25 ± 6.19	0.14	0.889
Depression level				
Mild depression (*n*, %)	19 (65.5)	18 (64.3)	0.009	0.922
Moderate to severe (*n*, %)	10 (34.5)	10 (35.7)		

### 3.2. Effect of MPST intervention on depression of the elderly in nursing homes

**Figure 3:**
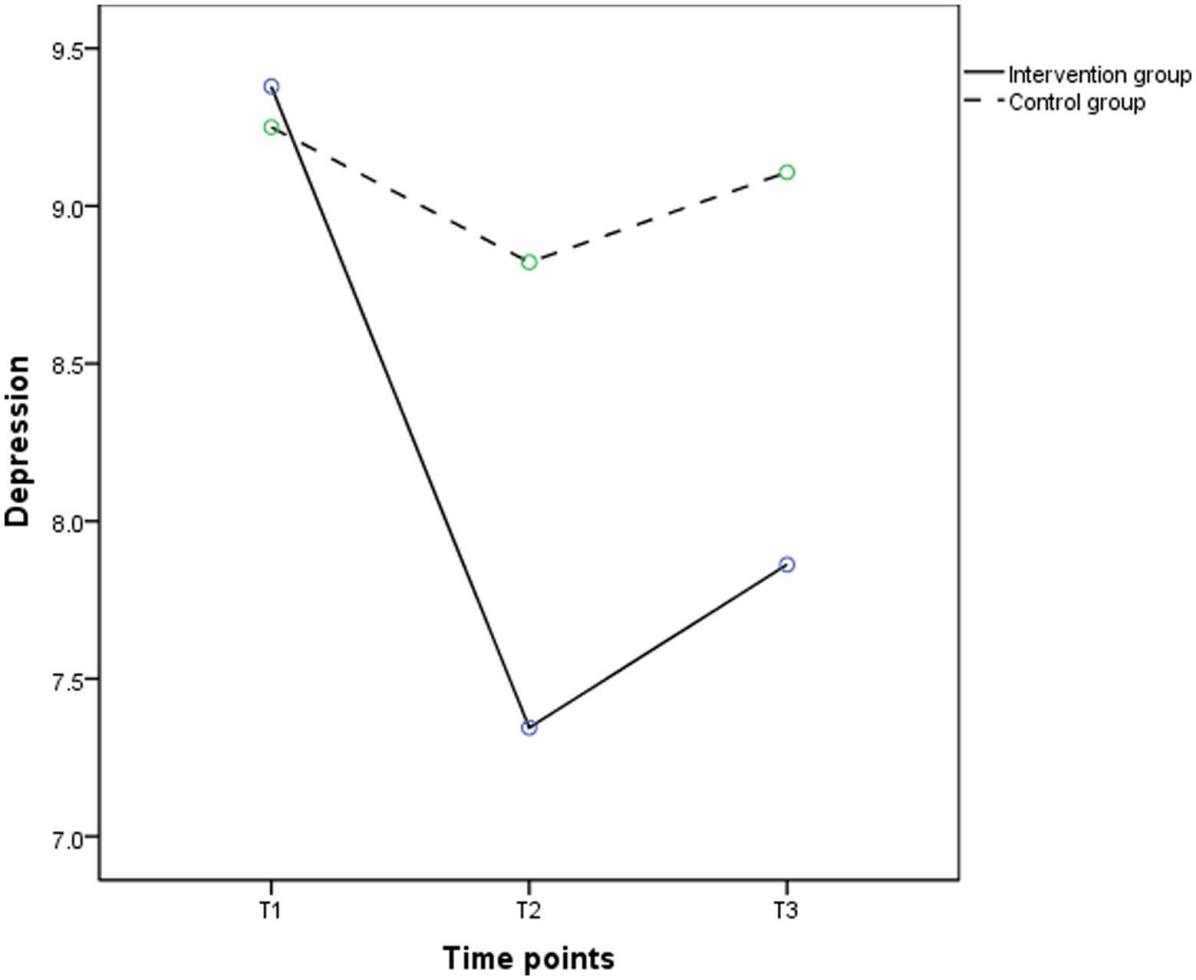
The trends in mean scores of depression in the intervention (solid line) and control groups (dashed line) between baseline and 3 months post intervention.

As shown in Table 4, the results of the repeated measures ANOVA showed that no differences in group effects (*F* = 2.738, *p* > 0.05) on depression were observed between the two groups, while time effects (*F* = 24.771, *p* < 0.001) and the group by time interaction effects (*F* = 11.831, *p* < 0.001) were significantly different. Figure 3 showed depression scores in the intervention group declined significantly from T1 to T2 and increased from T2 to T3, while depression scores in the control group did not change significantly from T1 to T3. Table 5 showed that participants in the intervention group had significant improvements in depressive symptoms at T2 (*t* = −2.698, *p* < 0.01) and T3 (*t* = −2.297, *p* < 0.05) compared to the control group, which indicates that depressive symptoms were significantly improved in the elderly receiving MPST compared to the control group and the effect was maintained at 3 months post-intervention.

**Table 4:** Analysis of the effect of time and intervention on depression, coping ability, and self-efficacy.

Items	Group effect		Time effect		Interaction effect	
	*F*	*P*	*F*	*P*	*F*	*P*
Depression	2.738	0.104	24.771	<0.001	11.831	<0.001
Positive coping	1.575	0.028	14.626	<0.001	16.305	<0.001
Negative coping	6.219	0.016	43.290	<0.001	24.580	<0.001
Self-efficacy	2.840	0.102	14.993	<0.001	18.033	<0.001

**Table 5:** Comparison of depression score among the intervention group and control group after MPST.

	Intervention group *n* = 29 (M ± SD)	Control group *n* = 28 (M ± SD)	*t*	*P*
T2	7.34 ± 2.08	8.82 ± 2.18	−2.698	0.009
T3	7.86 ± 2.18	9.11 ± 1.89	−2.297	0.025

### 3.3. Effect of MPST intervention on coping of the elderly in nursing homes

**Figure 4:**
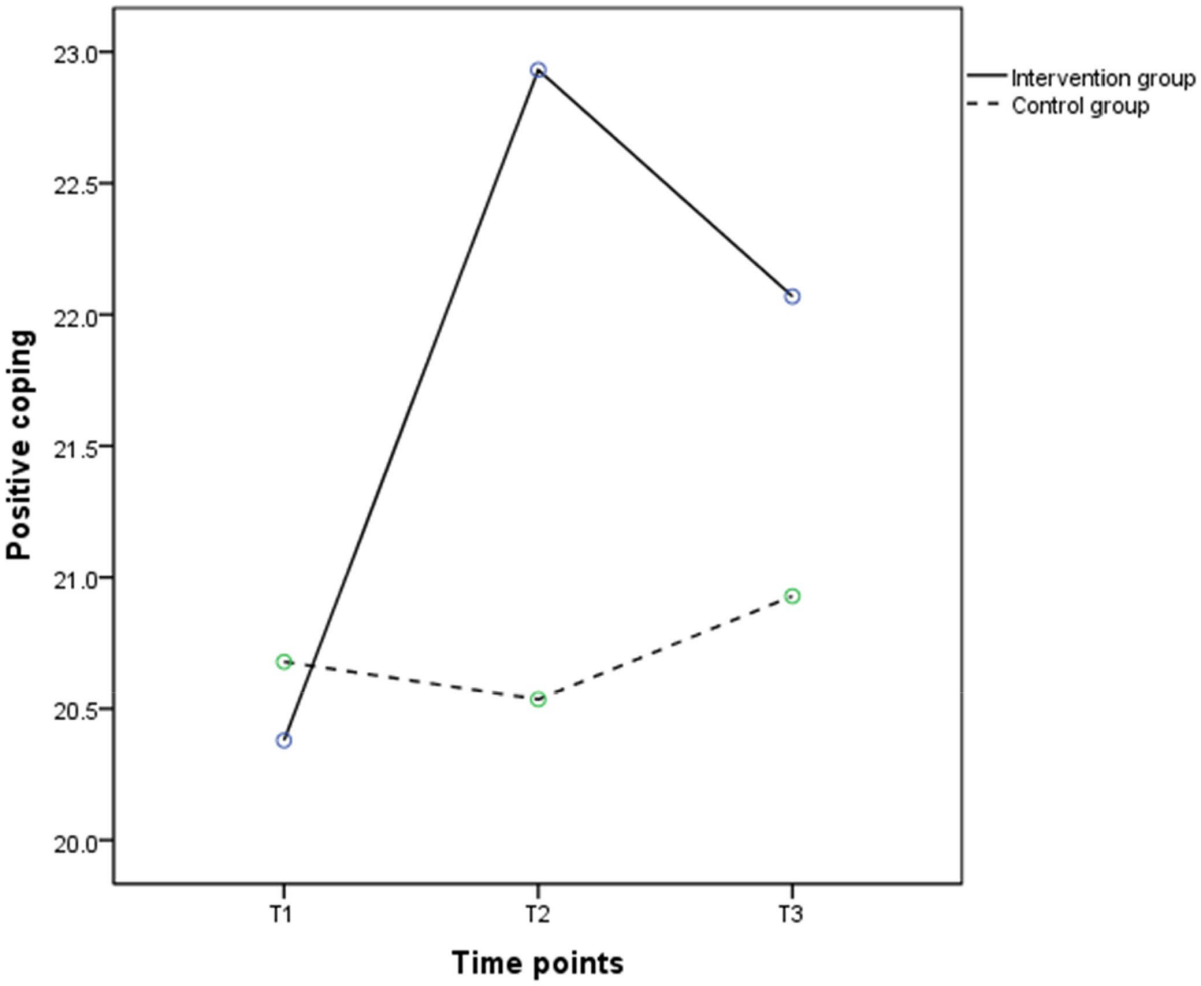
The trends in mean scores of positive coping in the intervention (solid line) and control groups (dashed line) between baseline and 3 months post intervention.

**Figure 5:**
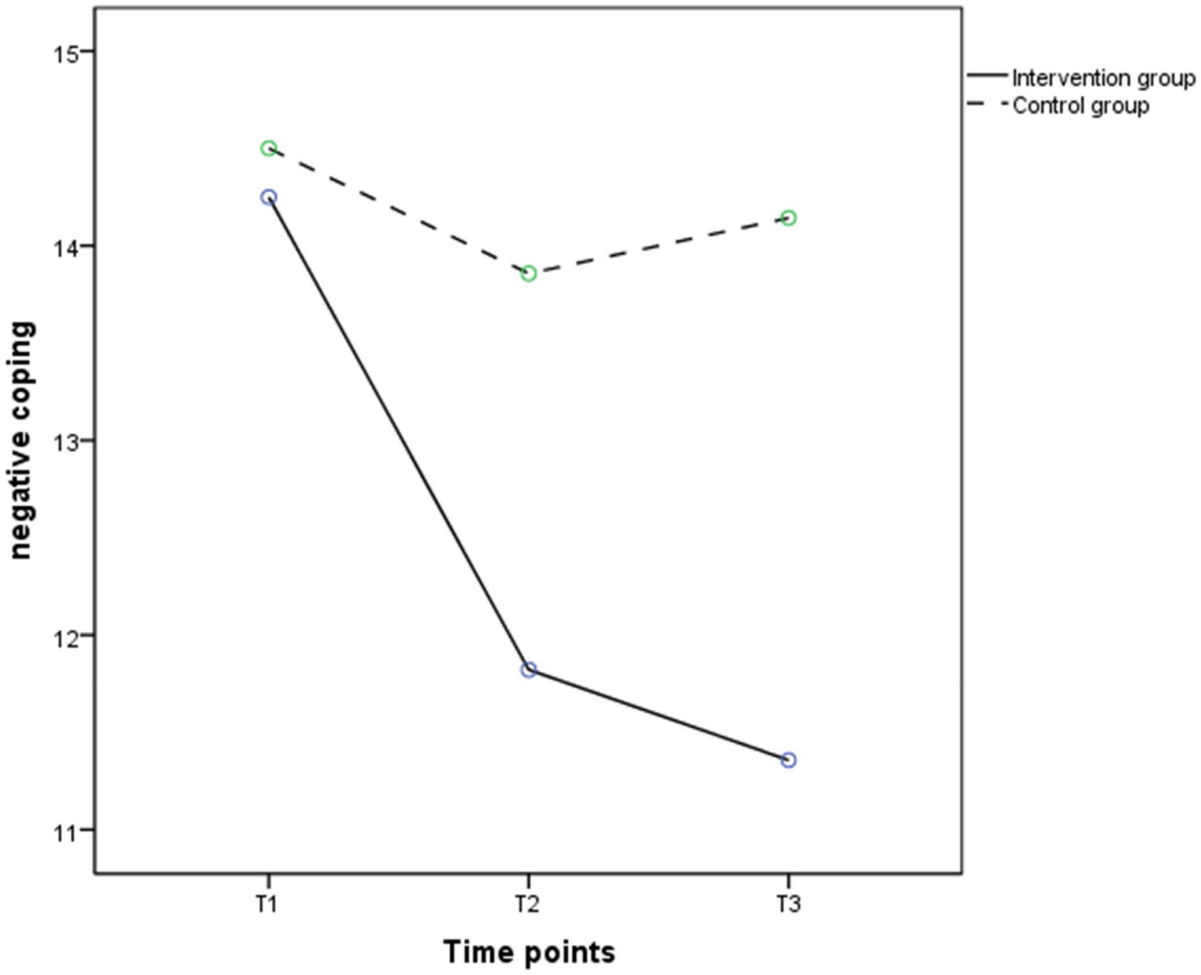
The trends in mean scores of negative coping in the intervention (solid line) and control groups (dashed line) between baseline and 3 months post intervention.

According to the results of repeated measure ANOVA, the group effects (*F* = 1.575, *p* < 0.05), time effects (*F* = 14.626, *p* < 0.001), and interaction effects (*F* = 16.305, *p* < 0.001) on positive coping were significant. Figure 4 shows that from T1 to T2, positive coping in the intervention group had a significant upward trend and slightly decreased in the control group; from T2 to T3, positive coping in the intervention group slowly decreased. As shown in Table 6, participants in the intervention group showed significant improvements in positive coping at T2 (*t* = − 2.744, *p* < 0.05) compared to the control group; though the positive coping score in the intervention group was higher than the control group at T3 (22.07 ± 3.89 vs. 20.93 ± 3.08), the difference between the two groups was not statistically significant (*p* > 0.05). As shown in Table 4, the group effects (*F* = 6.219, *p* < 0.05), time effects (*F* = 43.290, *p* < 0.001), and the group by time interaction effects (*F* = 24.580, *p* < 0.001) were significant in negative coping. Figure 5 showed negative coping in the intervention group consistently tended to decrease between T1 and T3, while there was no significant change in the control group. Table 7 showed that participants in the intervention group had significant reductions in depressive symptoms at T2 (*t* = −3.087, *p* < 0.01) and T3 (*t* = −3.799, *p* < 0.001) compared to the control group.

**Table 6:** Comparison of positive coping score among the intervention group and control group after MPST.

	Intervention group (*n* = 29)	Control group (*n* = 28)	*t*	*P*
T2	22.93 ± 3.31	20.54 ± 3.28	2.744	0.008
T3	22.07 ± 3.89	20.93 ± 3.08	1.224	0.226

**Table 7:** Comparison of negative coping score among the intervention group and control group after MPST.

	Intervention group (*n* = 29)	Control group (*n* = 28)	*t*	*P*
T2	11.83 ± 2.47	14.07 ± 3.01	−3.087	0.003
T3	11.31 ± 2.57	14.14 ± 3.05	−3.799	<0.001

### 3.4. Effect of MPST intervention on self-efficacy of the elderly in nursing homes

**Figure 6:**
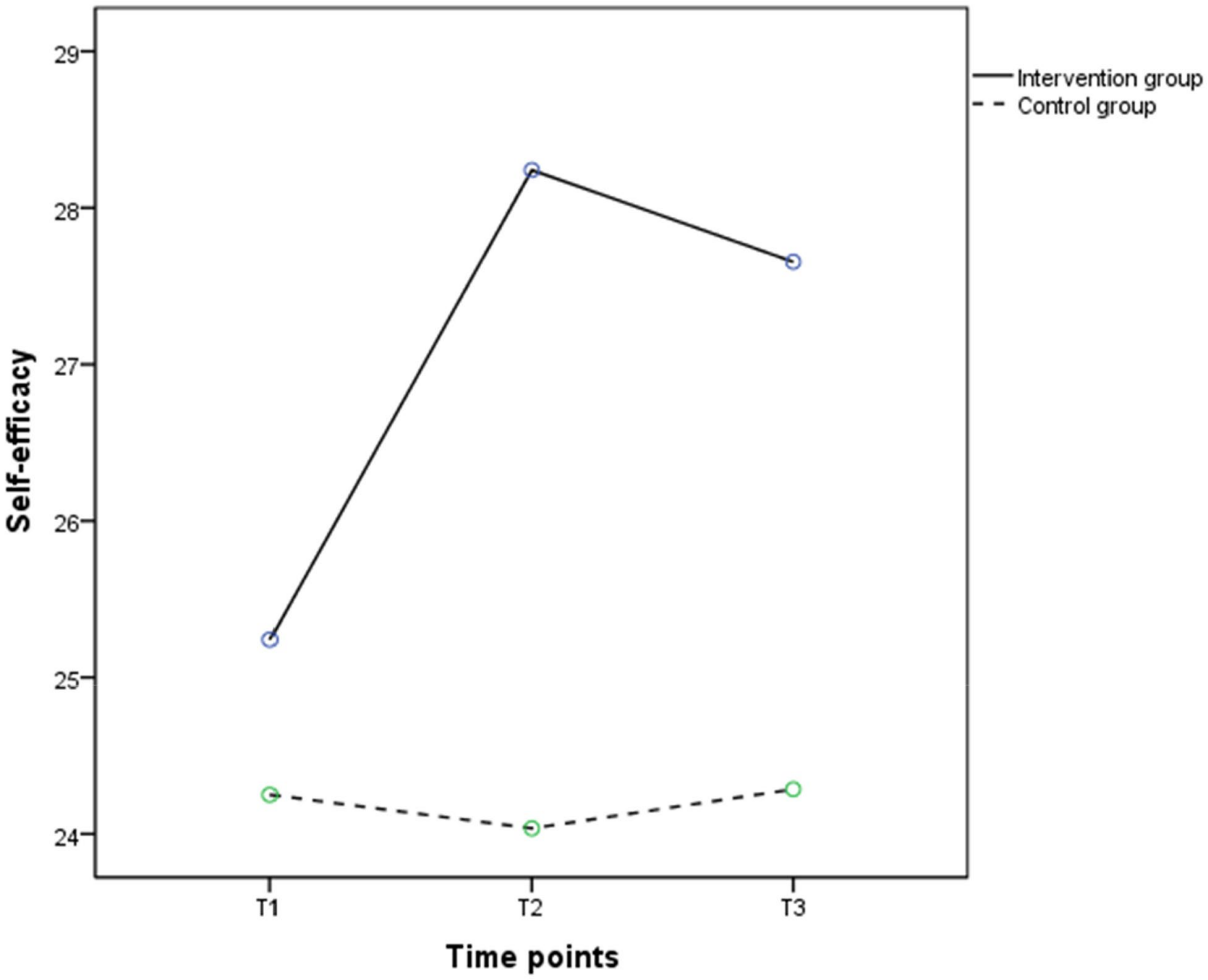
The trends in mean scores of self-efficacy in the intervention (solid line) and control groups (dashed line) between baseline and 3 months post intervention.

Table 4 shows that there were no significant differences in group effects (*F* = 2.840, *p* > 0.05) on self-efficacy between the two groups, while time effects (*F* = 14.993, *p* < 0.001) and the group by time interaction effects (*F* = 18.033, *p* < 0.001) were significantly different. Figure 6 shows that the self-efficacy of the intervention group rose to T2 and then decreased, while the control group showed insignificant changes between T1 and T3 (Table 8). The *t*-test results showed that the self-efficacy scores of the intervention group were higher than the control group at T2 (28.69 ± 6.66 vs. 24.57 ± 6.41) and T3 (27.66 ± 6.15 vs. 24.29 ± 6.07), which were statistically significance (*p* < 0.05).

**Table 8:** Comparison of self-efficacy score among the intervention group and control group after MPST.

	Intervention group (*n* = 29)	Control group (*n* = 28)	*t*	*P*
T2	28.69 ± 6.66	24.57 ± 6.41	2.376	0.021
T3	27.66 ± 6.15	24.29 ± 6.07	2.080	0.042

## 4. Discussion

This study investigated the effects of MPST on depression, coping ability, and self-efficacy of the elderly in nursing homes. The results found that the elderly who received MPST had reduced depression and increased levels of coping and self-efficacy.

In this study, participants in the intervention group had lower depression levels than the control group at the T2, indicating that MPST is superior to usual care in improving depression in older adults, consistent with the results of previous studies (Gellis et al., 2007, 2014). Moreover, some studies have confirmed that PST could apply to the elderly with depression in different treatment settings (Albert et al., 2019; Kanellopoulos et al., 2020), which reported the effectiveness of PST on depression in older adults under home care as well as outpatient follow-up. This study revealed the effectiveness of PST on depression in older adults who lived in the nursing home setting, consistent with the above findings. MPST can improve older adults’ misconceptions about daily hassles, reduces attitudes of avoiding problems and improves their problem-solving skills, which promotes stress reduction, enhances self-management confidence, and decrease depression (Shang et al., 2021). Separately, older adults receiving PST still maintained significantly lower depression than usual care at T3, demonstrating that the beneficial effects of MPST remained invariable at 3 months post-intervention.

Older adults in nursing homes are often exposed to frequent stressful events due to weakened social support and physical dysfunction, and the choice of coping style may more easily affect their mental health status (Fernández-Pérez et al., 2020; Bergmans et al., 2021). The present study indicated that the elderly who received MPST had higher positive coping abilities as well as lower negative coping abilities compared to usual care at T2, consistent with the result of Visser et al. (2016). Positive coping and depression possess a significant positive association and can influence each other. MPST provides the elderly with positive approaches to problem solving, leading to a sense of contentment in the process and increases positive coping, and indirectly improve depression in the elderly (Damush et al., 2016; Visser et al., 2016). While, positive coping in the intervention group was higher than the control group at T3, it was not statistically significant. On the contrary, negative coping in the intervention group remained significantly lower than that in the control group at T3. The reason for the different results may be that PST focuses on correcting negative problem orientation of older adults and targeting their negative behaviors with strategies (Choi et al., 2016; Bai et al., 2022). Therefore, MPST produced more impressive improvements in negative coping of older adults.

Self-efficacy is a subjective judgement of an individual’s own ability to behave judgment. Studies have revealed that the elderly with low self-efficacy have less confidence in handling daily events and less willing to partake in social activities, thus becoming lonely and depressed (Qin et al., 2020). Due to diminishing family support, changing interpersonal relationships, and adjustment to new environments, the elderly in nursing homes are prone to experience lower self-efficacy (Olsen et al., 2015; Könner et al., 2016). The results of this study showed that the self-efficacy of the intervention group at T2 and T3 were significantly different from those of the control group (both *p* < 0.05), indicating that MPST was effective in improving the self-efficacy of older adults in nursing homes, similar to the results of Rahim’s study (Habibi et al., 2016). This may be related to the ability of MPST to correct individuals’ misperceptions of problems and promote positive behavior change (Haejung et al., 2015; Graven et al., 2018). During the intervention, the researcher helped individuals to view problems correctly, learn problem-solving skills, and apply the skills to real problems to enhance individuals’ self-efficacy by solving problems and improving self-efficacy.

### 4.1. Evaluation of intervention programs

Considering the decreasing attention, memory and thinking of the elderly in nursing homes, MPST arranged theoretical knowledge sessions with examples of the elderly’s daily life to enhance their understanding of the relationship between “problems-depressive symptoms-physical functioning.” To ensure that older adults better understand, MPST includes question-and-answer section during in each session. When arranging the activity schedule, MPST uses symbols to represent different activities instead of written descriptions of daily activities to reduce the learning burden of older adults with lower education levels.

### 4.2. Limitation

Our study had several limitations. Firstly, the participants in this study were all from two nursing homes and the small sample size of this study may have an impact on the generalizability of the findings. Secondly, this study only used a depression screening scale to assess depression in older adults. Thirdly, MPST in this study was only compared with usual care and not with conventional PST. Finally, this study was only followed up to 3 months post-intervention, which could not assess the long-term effect of MPST on depression of older adults in nursing homes. Future studies with multicenter, large samples and extended follow-up could be conducted to determine the effect of the intervention on the improvement of depression in older adults.

## 5. Conclusion

The findings of this study suggest that MPST could be beneficial in reducing depressive symptoms and enhancing positive coping and self-efficacy levels in older adults in nursing homes, which provides an essential reference for mental health care of older adults in nursing homes.

## Data availability statement

The raw data supporting the conclusions of this article will be made available by the authors, without undue reservation.

## Ethics statement

The studies involving human participants were reviewed and approved by the Review Board of Xiangya School of Nursing, Central South University. The patients/participants provided their written informed consent to participate in this study.

## Author contributions

XW and JX made significant contributions in conceptual design, data acquisition, data analysis and interpretation. JL, CZ, XZ, XD, and HC conducted statistical analysis and provided consultation in the study design and the intervention development. YD, SW, ML, and QZ provided advice during the research design process and critically revised the contents of the manuscript. All authors contributed to the article and approved the submitted version.

## Funding

This research was supported by the Hunan Innovative Province Construction Project of Hunan Province (No. 2019SK2143).

## Conflict of interest

The authors declare that the research was conducted in the absence of any commercial or financial relationships that could be construed as a potential conflict of interest.

## Publisher’s note

All claims expressed in this article are solely those of the authors and do not necessarily represent those of their affiliated organizations, or those of the publisher, the editors and the reviewers. Any product that may be evaluated in this article, or claim that may be made by its manufacturer, is not guaranteed or endorsed by the publisher.
